# Ten-year outcomes of a randomised trial of laparoscopic versus open surgery for colon cancer

**DOI:** 10.1007/s00464-016-5270-6

**Published:** 2016-10-12

**Authors:** Charlotte L. Deijen, Jeanine E. Vasmel, Elly S. M. de Lange-de Klerk, Miguel A. Cuesta, Peter-Paul L. O. Coene, Johan F. Lange, W. J. H. Jeroen Meijerink, Jack J. Jakimowicz, Johannes Jeekel, Geert Kazemier, Ignace M. C. Janssen, Lars Påhlman, Eva Haglind, H. Jaap Bonjer, On behalf of the COLOR (COlon cancer Laparoscopic or Open Resection) study group, R. Hellberg, E. Haglind, G. Kurlberg, P. G. Lindgren, E. Lindholm, L. Påhlman, M. Dahlberg, Y. Raab, B. Anderberg, S. Ewerth, M. Janson, J. E. Åkerlund, K. Smedh, A. Montgomery, S. Skullman, P. O. Nyström, A. Kald, A. Wänström, J. Dàlen, I. Svedberg, G. Edlund, U. Kressner, A. N. Öberg, O. Lundberg, G. E. Lindmark, T. Heikkinen, M. Morino, G. Giraudo, A. M. Lacy, S. Delgado, E. Macarulla Sanz, J. Medina Díez, O. Schwandner, T. H. Schiedeck, H. Shekarriz, C. Bloechle, I. Baca, O. Weiss, S. Msika, G. Desvignes, K. L. Campbell, A. Cuschieri, H. J. Bonjer, W. R. Schouten, G. Kazemier, J. F. Lange, E. van der Harst, P. P. L. O. Coene, P. Plaisier, M. J. O. E. Bertleff, M. A. Cuesta, W. van der Broek, W. J. H. J. Meijerink, J. J. Jakimowicz, G. Nieuwenhuijzen, J. Maring, J. Kivit, I. M. C. Janssen, E. J. Spillenaar-Bilgen, F. Berends

**Affiliations:** 1 Department of Surgery, VU University Medical Centre, De Boelelaan 1117, 1081 HV Amsterdam, The Netherlands; 2 Department of Surgery, Maasstad Hospital, Rotterdam, The Netherlands; 3 Department of Surgery, Erasmus Medical Centre, Rotterdam, The Netherlands; 4 Department of Design Engineering, Delft University of Technology, Delft, The Netherlands; 5 Department of Surgery, Rijnstate Hospital, Arnhem, The Netherlands; 6 Department of Surgery, Uppsala University Hospital, Uppsala, Sweden; 7 Department of Surgery, Sahlgrenska University Hospital/Östra, Gothenburg, Sweden

**Keywords:** Colon cancer, Surgery, Laparoscopic, Treatment, Randomised clinical trial

## Abstract

**Background:**

Laparoscopic surgery for colon cancer is associated with improved recovery and similar cancer outcomes at 3 and 5 years in comparison with open surgery. However, long-term survival rates have rarely been reported. Here, we present survival and recurrence rates of the Dutch patients included in the COlon cancer Laparoscopic or Open Resection (COLOR) trial at 10-year follow-up.

**Methods:**

Between March 1997 and March 2003, patients with non-metastatic colon cancer were recruited by 29 hospitals in eight countries and randomised to either laparoscopic or open surgery. Main inclusion criterion for the COLOR trial was solitary adenocarcinoma of the left or right colon. The primary outcome was disease-free survival at 3 years, and secondary outcomes included overall survival and recurrence. The 10-year follow-up data of all Dutch patients were collected. Analysis was by intention-to-treat. The trial was registered at ClinicalTrials.gov (NCT00387842).

**Results:**

In total, 1248 patients were randomised, of which 329 were Dutch. Fifty-eight Dutch patients were excluded and 15 were lost to follow-up, leaving 256 patients for 10-year analysis. Median follow-up was 112 months. Disease-free survival rates were 45.2 % in the laparoscopic group and 43.2 % in the open group (difference 2.0 %; 95 % confidence interval (CI) −10.3 to 14.3; *p* = 0.96). Overall survival rates were 48.4 and 46.7 %, respectively (difference 1.7 %; 95 % CI −10.6 to 14.0; *p* = 0.83). Stage-specific analysis revealed similar survival rates for both groups. Sixty-two patients were diagnosed with recurrent disease, accounting for 29.4 % in the laparoscopic group and 28.2 % in the open group (difference 1.2 %; 95 % CI −11.1 to 13.5; *p* = 0.73). Seven patients had port- or wound-site recurrences (laparoscopic *n* = 3 vs. open *n* = 4).

**Conclusions:**

Laparoscopic surgery for non-metastatic colon cancer is associated with similar rates of disease-free survival, overall survival and recurrences as open surgery at 10-year follow-up.

**Electronic supplementary material:**

The online version of this article (doi:10.1007/s00464-016-5270-6) contains supplementary material, which is available to authorized users.

Laparoscopic surgery for colon cancer has proven to result in short-term benefits compared to open surgery, such as reduced blood loss, less post-operative pain and shorter length of hospital stay [1–3]. However, there are few studies on long-term outcomes of laparoscopic and open surgery for colon cancer [4–7]. This is remarkable, as malignancy of the colon and rectum is the third most common malignancy worldwide, accounting for approximately 1,361,000 new patients and 694,000 deaths every year [8].

Early detection of recurrent colon cancer is important because prompt management of these recurrences is associated with improved survival [9]. Current colon cancer guidelines advocate follow-up up to 5 years after surgery. [9, 10] Nevertheless, knowledge of the course of disease beyond the period of 5 years is limited. Therefore, it remains unclear whether limiting follow-up to 5 years post-operatively is sufficient.

The COlon cancer Laparoscopic or Open Resection (COLOR) trial was designed as an international multicentre randomised trial to demonstrate non-inferiority of laparoscopic surgery for colon cancer compared with the conventional open resection [11]. Previously, 3- and 5-year results have been published, and similar survival outcomes for both groups were reported [12]. Here, we present the long-term outcomes of Dutch patients included in the COLOR trial at 10-year follow-up.

## Materials and methods

### Study design

The COLOR trial is a randomised, non-inferiority, open-label trial. Between March 1997 and March 2003, patients were recruited by 29 hospitals in eight countries. The trial was approved by the ethics committee of each participating hospital. Because 10-year follow-up was not included in the initial COLOR trial protocol, the study had to be re-opened in all participating countries separately. The Netherlands is a relative small country and its geography made it possible to accurately check all medical records and collect all data. Therefore, only data of Dutch patients were used for this study. The Ethical Committee of VUmc approved 10-year follow-up of all Dutch patients. This trial is registered at ClinicalTrials.gov, number NCT00387842.

### Participants

The main criterion for inclusion in the COLOR trial was non-metastatic solitary adenocarcinoma of the caecum, ascending colon, descending colon, or sigmoid colon above the peritoneal deflection. Tumours of the transverse colon or splenic flexure were not included in this study because laparoscopic surgery of these tumours was considered technically more challenging and prone to conversion to open surgery. Diagnosis was to be made by barium enema radiography or colonoscopy. A biopsy was required in polyps, not in macroscopically evident adenocarcinomas. Metastatic disease was excluded by radiological imaging of the chest and liver. Exclusion criteria were: body mass index >30, distant metastases, multiple primary colon tumours, invasion of adjacent structures, signs of obstruction, previous ipsilateral surgery of the colon, history of malignant disease (with the exception of curative treatment for basal cell carcinoma of the skin or in situ carcinoma of the cervix) and absolute contraindication for general anaesthesia or pneumoperitoneum. All patients gave written informed consent.

### Randomisation and masking

Eligible patients were assigned to either laparoscopic resection or open resection at random in a 1:1 ratio and stratified according to participating centre and type of resection. Randomisation was performed by the trial coordinator (RV, who was succeeded by EK) at Erasmus University Medical Center, Rotterdam, Netherlands, and allocation was performed by telephone or fax. Neither patients nor caregivers were blinded to the result of randomisation.

### Procedures

Patients in both groups had the same extent of resections: in right hemicolectomy a resection of the caecum, ascending colon and hepatic flexure, in left hemicolectomy a resection with a margin of at least 5 cm below and 5 cm above the lesion and in sigmoidectomy a resection of the sigmoid of at least 5 cm below and 5 cm above the lesion. Pre- and post-operative care and adjuvant treatment were applied according to local protocols.

Follow-up for both groups was required at least once a year during the first 5 post-operative years and included colon, liver and thorax imaging studies at 3-year follow-up. After 5 years, further follow-up was at the surgeon’s discretion. Participating centres treated detected recurrences according to local protocols, including resection and chemotherapy.

Surgical teams had performed at least 20 laparoscopically assisted colectomies and had to submit an unedited videotape of a laparoscopically assisted colectomy to assess safety of surgical techniques before entering the trial. Patients in the laparoscopic group could be converted preoperatively to an open resection if there was malfunctioning equipment or if no surgeon with laparoscopic skills was available. All converted patients, i.e. preoperative and intraoperative, remained in the laparoscopic group for analysis based on intention-to-treat principle.

### Outcomes

The primary outcome was disease-free survival at 3 years, which has been reported earlier [12]. Secondary outcomes included overall survival and pattern of recurrence. Recurrences were defined as local or distant. Furthermore, we defined local recurrence as recurrence at the surgical site or port- or wound-site and distant recurrence as all other recurrences. When no clinical signs of recurrence were present at 10-year follow-up, further imaging was not done and the patient was considered as not having recurrent disease. For 10-year follow-up results, data of all Dutch patients were collected. The hospital information system was used to collect details at 10 years after index surgery and when no information was available, the general practitioner of the patient was consulted. If information about survival of the patients was missing, the Municipal Personal Records Database was checked.

### Statistical analyses

Analyses were performed according to the intention-to-treat principle. Baseline characteristics were compared by using Student’s *t* test or a Mann–Whitney *U* test for numerical variables and a Chi-square test or an exact test where necessary. The Kaplan–Meier method was used to calculate the median follow-up period [13] and 10-year disease-free survival, overall survival and recurrence rates. Survival was calculated as time from surgery to last date of follow-up or date of death. IBM SPSS version 22 was used for statistical analyses.

## Results

### Patients

**Fig. 1:**
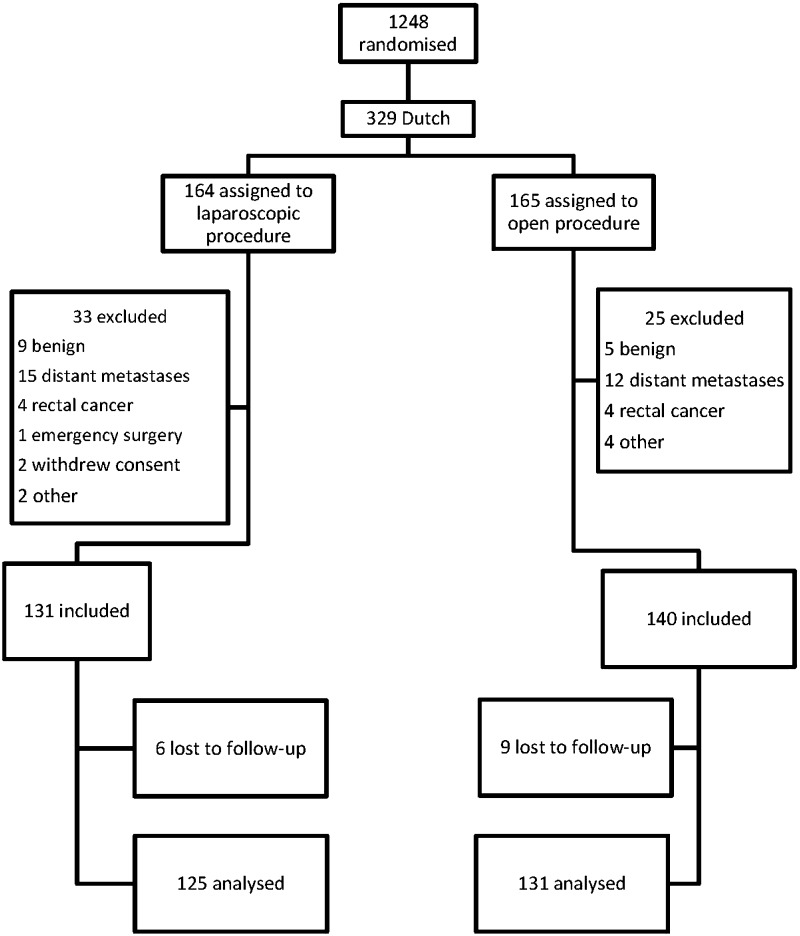
Trial profile

In total, 1248 patients were randomly assigned to either laparoscopic resection or conventional open resection, of which the six participating Dutch centres recruited 329. Of the Dutch patients, 58 were excluded for various reasons (Fig. 1). The first patient inclusion in the Netherlands was at 21 March 1997 and the last at 10 March 2003. In November 2014 collecting of 10-year follow-up data was started. In the laparoscopic and open group, six and nine patients, respectively, had been lost to follow-up, leaving 256 patients for 10-year analysis. Of those, 125 patients were assigned to be operated laparoscopically and 131 patients to be operated through an open procedure (Fig. 1). The median follow-up of all patients was 112 months in the laparoscopic group (range 0.03–198.92) and 111 months in the open group (range 0.10–194.89) (*p* = 0.83). Median follow-up of survivors was 156 months in the laparoscopic group (range 117.97–198.92) and 150 months in the open group (range 105.11–194.13).

Baseline characteristics showed no significant differences between the two groups (Table 1). Operative and pathological data showed no differences except for length of operative procedure, which was longer in the laparoscopic group (140 vs. 95 min, *p* < 0.001) and blood loss, which was less in the laparoscopic group (113 vs. 200 mL, *p* = 0.02). Distribution of disease stage and size of tumour was similar in both groups (Table 2).

**Table 1:** Patient baseline characteristics

	Laparoscopic colectomy (*n* = 131)	Open colectomy (*n* = 140)	Total (*n* = 271)
Age (years), median (range)	71 (54–84)	72 (54–84)	71 (54–84)
Gender, *n* (%)			
Male	63 (48.1)	76 (54.3)	139 (51.3)
Female	68 (51.9)	64 (45.7)	132 (48.7)
ASA group, *n* (%)			
I	44 (33.6)	49 (35.0)	93 (34.3)
II	62 (47.3)	73 (52.1)	135 (49.8)
III	20 (15.3)	18 (12.9)	38 (14.0)
Missing data	5 (3.8)	–	5 (1.8)
Body mass index (kg/m^2^), median (range)	24.8 (20.2–29.6)	25.1 (20.2–30.7)	24.9 (20.2–29.9)

*Range* 10th to 90th percentile, *ASA* American society of anesthesiologists classification

**Table 2:** Operative and pathological data

	Laparoscopic colectomy (*n* = 125)	Open colectomy (*n* = 131)	Total (*n* = 256)	*p* value
Intervention, *n* (%)				0.527^a^
Right hemicolectomy	68 (54.4)	66 (50.4)	134 (52.3)	
Left hemicolectomy	10 (8.0)	10 (7.6)	20 (7.8)	
Sigmoidectomy	41 (32.8)	52 (39.7)	93 (36.3)	
Other	6 (4.8)	3 (2.3)	9 (3.5)	
Conversions				
Preoperative	6 (4.8)	–		
Intraoperative	34 (27.2)	–		
Duration of intervention (min), median (range)				
In theatre	180 (130–270)	135 (93.5–210)		<0.001^b^
Skin to skin	140 (95–229.5)	95 (70–160.2)		<0.001^b^
Blood loss (ml), median (range)	112.5 (13.5–559)	200 (50–825)		0.024^b^
Size of tumour (cm), median (range)	4.0 (2.0–6.8)	4.0 (2.5–7.5)		0.168^c^
Resection margins, *n* (%)				1.00^a^
Positive	1 (0.8)	2 (1.5)		
Negative	117 (99.2)	128 (98.5)		
Tumour stage, *n* (%)				0.974^a^
I	31 (25.0)	30 (23.4)	61 (24.2)	
II	54 (43.5)	57 (44.5)	111 (44.0)	
III	39 (31.5)	41 (32.0)	80 (31.7)	

*Range* 10th to 90th percentile

^a^Fisher’s exact test

^b^Mann–Whitney *U* test

^c^Student’s *t* test

### Conversion

Of 125 patients who were assigned to undergo a laparoscopic procedure, conversion to open surgery was performed in 40 patients (32 %). In six patients, the decision for conversion was made preoperatively (poor cardiac condition (*n* = 3), randomisation error (*n* = 1), extensive T4 tumour (*n* = 1) and unknown (*n* = 1)). In 34 patients (27 %), conversion was performed during the operation, reasons for conversion were fixation of the tumour (*n* = 10), adhesions (*n* = 3), the tumour could not be identified (*n* = 8), macroscopic metastases were found (*n* = 2), other reasons (*n* = 10), and in one patient, the reason was unknown.

### Disease-free survival

**Fig. 2:**
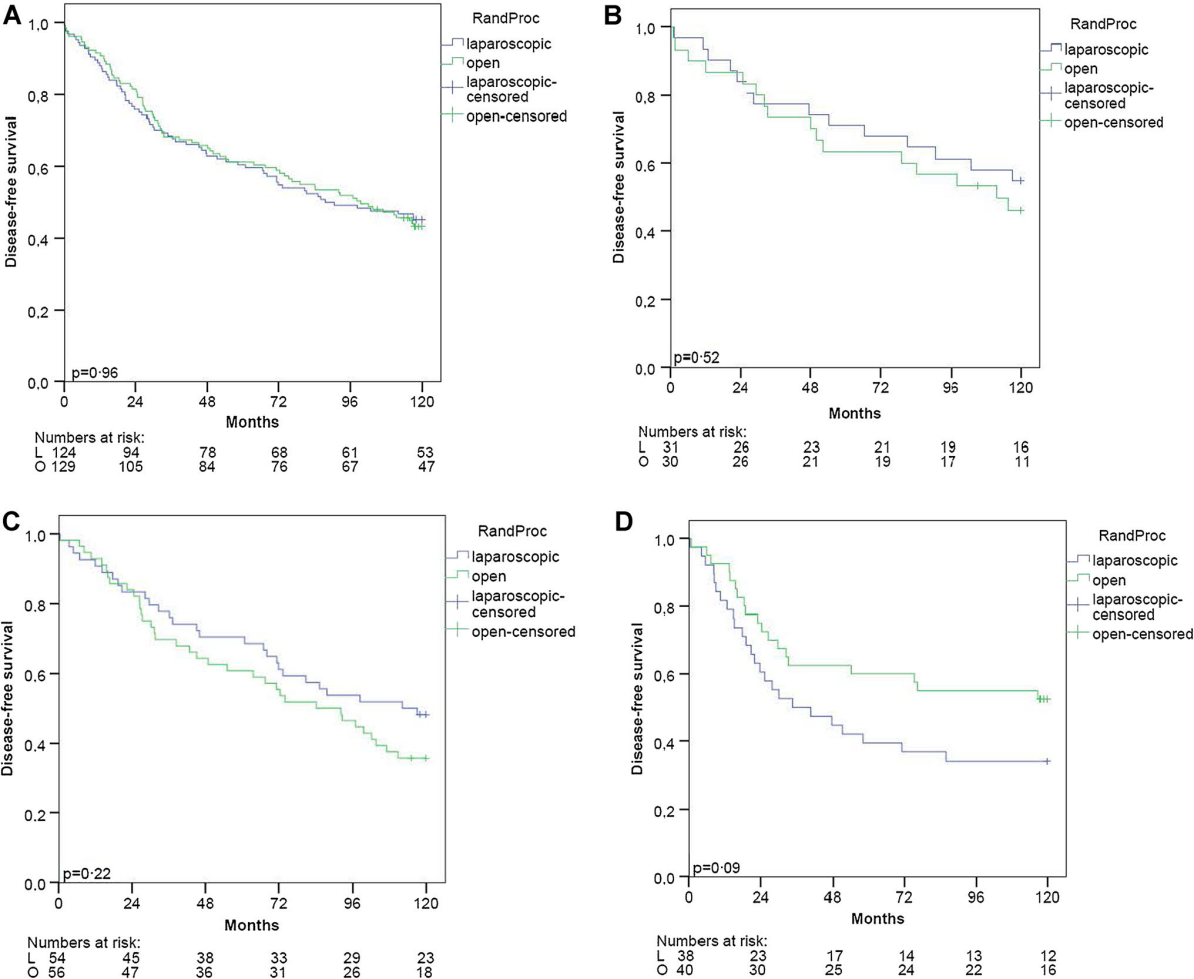
Disease-free survival. **A** All stages, **B** stage I, **C** stage II and **D** stage III

The disease-free survival rate at 10 years post-operatively was 45.2 % in the laparoscopic group and 43.2 % in the open group (difference 2.0 %; 95 % confidence interval (CI) −10.3 to 14.3; *p* = 0.96). In patients with stage I colon cancer, disease-free survival rates were 54.8 and 45.9 % for the laparoscopic and open group, respectively (difference 8.9 %; 95 % CI −16.2 to 34.0; *p* = 0.52). In patients with stage II disease, these rates were 48.1 and 35.7 % (difference 12.4 %; 95 % CI −5.9 to 30.7; *p* = 0.22) and in patients with stage III disease 34.2 % in the laparoscopic group and 52.5 % in the open group (difference −18.3 %; 95 % CI −39.9 to 3.3; *p* = 0.09) (Fig. 2).

### Overall survival

At 10-year follow-up, 133 patients had died, 64 in the laparoscopic group and 69 in the open group. Fifty-three patients who died had recurrent disease (27 patients in the laparoscopic group and 26 patients in the open group). The 10-year overall survival rate was 48.4 % in the laparoscopic group and 46.7 % in the open group (difference 1.7 %; 95 % CI −10.6 to 14.0; *p* = 0.83). In patients with stage I colon cancer, overall survival rates were 58.1 and 52.7 % for the laparoscopic and open group, respectively (difference 5.4 %; 95 % CI −19.7 to 30.5; *p* = 0.67). In patients with stage II disease, these rates were 51.9 and 41.1 % (difference 10.8 %; 95 % CI −7.8 to 29.4; *p* = 0.23) and in patients with stage III disease 36.8 % in the laparoscopic group and 50.8 % in the open group (difference −14.0 %; 95 % CI −35.8 to 7.8; *p* = 0.22) (Figure as Supplementary material).

### Recurrences

**Fig. 3:**
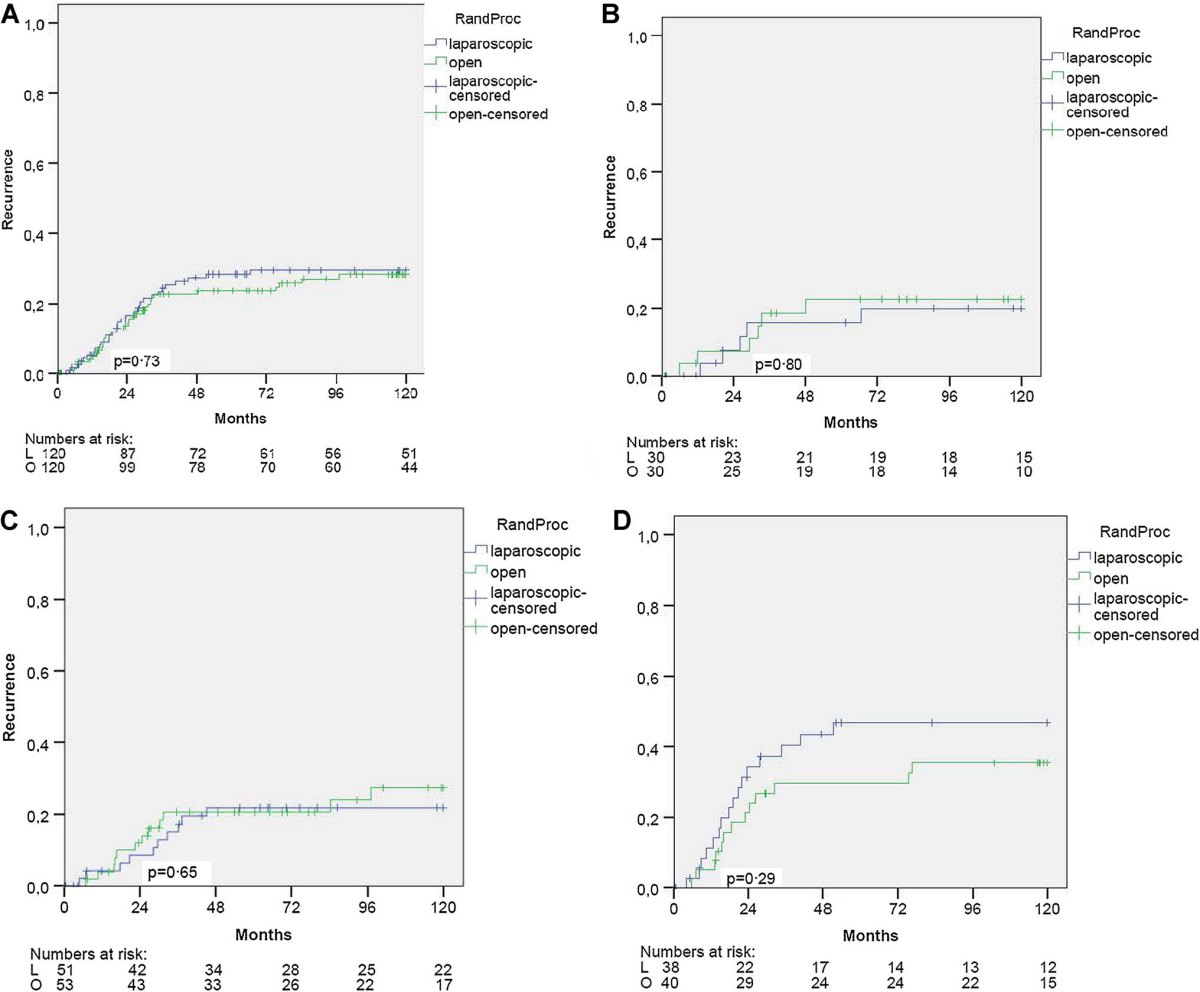
Recurrence. **A** All stages, **B** stage I, **C** stage II and **D** stage III

A total of 62 patients developed recurrent disease during the 10-year follow-up period, accounting for a recurrence rate of 29.4 % in the laparoscopic group and 28.2 % in the open group (difference 1.2 %; 95 % CI −11.1 to 13.5; *p* = 0.73). In patients with stage I colon cancer, recurrence rates were 19.8 and 22.5 % for the laparoscopic and open group, respectively (difference −2.7 %; 95 % CI −25.2 to 19.8; *p* = 0.80). In patients with stage II disease, these rates were 21.8 and 27.3 % (difference −5.5 %; 95 % CI −23.7 to 12.7; *p* = 0.65) and in patients with stage III disease 46.8 % in the laparoscopic group and 35.4 % in the open group (difference 11.4 %; 95 % CI −11.6 to 34.4; *p* = 0.29) (Fig. 3).

The site of recurrence did not significantly differ between the two groups. In total, 43 patients suffered a locoregional recurrence, 23 patients in the laparoscopic group and 20 patients in the open group. Seven patients had a recurrence in the port- or wound-site, three patients in the laparoscopic and four patients in the open group. The time of occurrence of the port- and wound-site recurrences after surgery was in the laparoscopic group 8.1, 30.9 and 34.7 months and in the open group 16.0, 16.7, 27.5 and 31.2 months. In total, 40 patients were diagnosed with a distant recurrence (19 in the laparoscopic and 21 in the open group), accounting for 69 distant recurrences (Table as Supplementary material).

At 5 years follow-up, 154 patients were alive and free of disease. Between 5 and 10 years after surgery five of these 154 patients (3 %) developed a first recurrence. Three other patients developed a recurrence between 5 and 10 years after surgery as well, and however, in these patients it was not the first recurrence.

## Discussion

The survival and recurrence rates 10 years after either laparoscopic or open colectomy for cancer are similar. At 10 years after surgery for stage I, II and III colon cancer, disease-free survival rates were 45.2 and 43.2 % in, respectively, the laparoscopic and open group. Overall survival rates were 48.4 and 46.7 % for the laparoscopic and open group. Lacy et al. [4] reported in 219 patients with colon cancer stage I–III at a median follow-up of 95 months similar cancer-free survival and overall survival rates between the laparoscopic and open groups as well. Due to reduction in surgical trauma, minimally invasive surgery was expected to be associated with improved oncological outcomes [14]. However, this assumption has not been validated by current available evidence.

Only 3 % of all patients that were free of disease and alive at 5 years developed a recurrence more than 5 years after index surgery. Merely two other studies on long-term survival after colon cancer surgery have been published. Similar patterns of recurrences were reported, but exact numbers were not provided [4, 5]. Hence, the current colon cancer guidelines recommendation to cease follow-up after 5 years after surgery appears justified.

The intraoperative conversion rate of this substudy was 27 %, which is higher than the 17 % overall intraoperative conversion rate of the COLOR trial. Other large randomised trials reported conversion rates of 11, 15, 21 and 25 % [3, 15–17]. All these trials were conducted between 1993 and 2005. In those years, routine preoperative imaging of colonic cancer was limited in most patients to barium enema and ultrasonography of the liver [2, 14, 15]. In the COLOR trial, imaging of the tumour was performed with computed tomography (CT) in 4 % of the patients and with barium enema in 40 % of the patients. In 81 % of the patients, a colonoscopy was done with tattooing of the tumour in 3 % [2]. Nowadays, abdominal CT has become a standard component of the diagnostic workup in patients with colon cancer allowing preoperative identification of patients with large and invasive colonic carcinomas which are not amenable to laparoscopic surgery. In this study, the reason for conversion was fixation of the tumour in one-third of the patients, and in one-fourth, the tumour could not be properly identified during the procedure, both as result of limitations in preoperative workup at the time this trial was conducted. The high rate of converted procedures may have been caused by limited technical skills among the surgeons, as well as deficiencies in the workup at that time, such as quality of the CT scan and lack of inking of the tumour at endoscopy, which was not part of the standard preoperative procedure at that time.

Even though in this report all converted patients were analysed in the laparoscopic group according to the intention-to-treat principle, survival rates of the laparoscopic and open group did not differ. However, the impact of conversion on survival remains unclear. A recent report on 104,400 patients included in the American National Cancer Database concluded that conversion from laparoscopic to open surgery did not result in compromised oncologic outcomes [18]. On the contrary, the CLASICC trial showed worse overall survival in converted patients at a median follow-up of 63 months [5].

Deposits of tumour cells at trocar sites (port-site metastases) were reported during the initial experience with laparoscopic colectomy for cancer [19, 20]. These findings stalled implementation of laparoscopic surgery in the management of colon cancer for more than a decade. In this study, cancer recurrences in the abdominal wall were noted within 10 years after surgery in 2 % of patients. All these recurrences occurred within 3 years after index surgery. In the CLASICC trial, 12 out of 641 (1.9 %) analysed patients had one or more port- or wound-site recurrences, ten (2.3 %) in the laparoscopic group and two (0.9 %) in the open group, without a significant difference [5]. The COST trial reported among 863 analysed patients, surgical wound metastases as first site of recurrence in four patients (0.9 %) in the laparoscopic group and two patients (0.5 %) in the open group at 5 years [21].

This report has several limitations. Firstly, follow-up until 10 years after index surgery was not part of the original protocol for the COLOR trial. This report only involves the Dutch patients of the COLOR trial representing one quarter of the entire study population. Although only a subgroup of patients was included, this study on long-term outcomes after colon cancer surgery involves one of the largest cohorts of patients reported to date. Furthermore, the primary outcome of the original study was disease-free survival at 3 years. This study was not powered for a 10-year follow-up period, and the number of patients must have been larger according to an adequate power analysis. Therefore, results as the high conversion rate of the Dutch population compared to the entire cohort should be interpreted with caution.

In conclusion, disease-free survival, overall survival and recurrence rates at 10-year follow-up after laparoscopic and open resection of non-metastatic and non-invasive colon cancer were similar.

## Electronic supplementary material

Below is the link to the electronic supplementary material.

Supplementary material 1 (DOCX 9 kb)

Supplementary material 2 (JPEG 123 kb)

Supplementary material 3 (DOCX 14 kb)
