# Supplementary material for: Ten-year outcomes of a randomised trial of laparoscopic versus open surgery for colon cancer
Source: Surg Endosc. 2016 Oct 12;31(6):2607–15. doi: 10.1007/s00464-016-5270-6 (PMC5443846; doi:10.1007/s00464-016-5270-6)
Supplement: Supplementary file 1 — Supplementary material 1 (DOCX 9 kb) [file 464_2016_5270_MOESM1_ESM.docx]

Supplementary figure

Overall survival

a. All stages

b. Stage I

c. Stage II

d. Stage III
