# Supplementary figures and images for: Ten-year outcomes of a randomised trial of laparoscopic versus open surgery for colon cancer
Source: Surg Endosc. 2016 Oct 12;31(6):2607–15. doi: 10.1007/s00464-016-5270-6 (PMC5443846; doi:10.1007/s00464-016-5270-6)

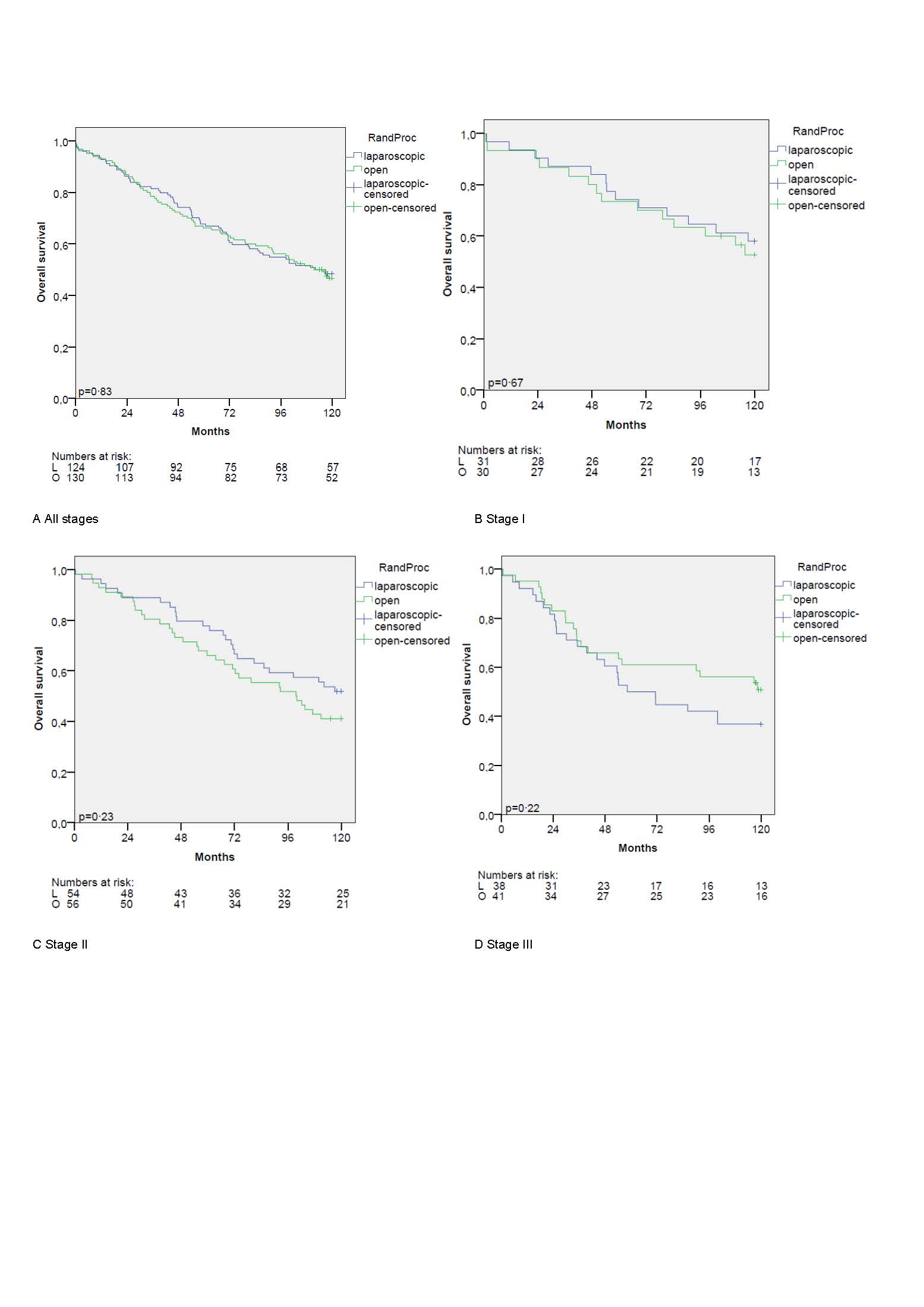

Supplement: Supplementary file 2 — Supplementary material 2 (JPEG 123 kb) [file 464_2016_5270_MOESM2_ESM.jpg]
