# Supplementary material for: Ten-year outcomes of a randomised trial of laparoscopic versus open surgery for colon cancer
Source: Surg Endosc. 2016 Oct 12;31(6):2607–15. doi: 10.1007/s00464-016-5270-6 (PMC5443846; doi:10.1007/s00464-016-5270-6)
Supplement: Supplementary file 3 — Supplementary material 3 (DOCX 14 kb) [file 464_2016_5270_MOESM3_ESM.docx]

|  | **Laparoscopic colectomy** | **Open colectomy** | **Overall** |
| --- | --- | --- | --- |
| **Number of total recurrences** |  |  |  |
| Locoregional | 23 | 20 | 43 |
| *Peritoneum* | 3 | 2 | 5 |
| *Primary tumour site* | 17 | 14 | 31 |
| *Port- or wound-site* | 3 | 4 | 7 |
| Liver | 15 | 19 | 34 |
| Lung | 7 | 11 | 18 |
| Adnex | 2 | 2 | 4 |
| Ossal | 2 | 3 | 5 |
| Brain | 0 | 1 | 1 |
| Spleen | 1 | 1 | 2 |
| Omentum | 2 | 0 | 2 |
| Lymph nodes | 2 | 0 | 2 |
| Pancreas | 1 | 0 | 1 |
| **Total** | **55** | **57** | **112** |

***Table: Pattern of recurrences in 62 patients***
